# Cdc42 regulates reactive oxygen species production in the pathogenic yeast *Candida albicans*

**DOI:** 10.1016/j.jbc.2021.100917

**Published:** 2021-06-25

**Authors:** Griffin P. Kowalewski, Asia S. Wildeman, Stéphanie Bogliolo, Angelique N. Besold, Martine Bassilana, Valeria C. Culotta

**Affiliations:** 1 Department of Biochemistry and Molecular Biology, Johns Hopkins University Bloomberg School of Public Health, Baltimore, Maryland, USA; 2 Université Côte d'Azur, CNRS, INSERM, Institute of Biology Valrose (iBV), Parc Valrose, Nice, France

**Keywords:** reactive oxygen species, yeast physiology, NADPH oxidase, fungi, morphogenesis, CMAC, 7-amino-4-chloromethylcoumarin, FBS, fetal bovine serum, Fre8-dTom, Fre8 to dTomato, HBSS, Hanks buffered saline solution, H_2_O_2_, hydrogen peroxide, IMDM, Iscove's modified Dulbecco's medium, NBT, nitroblue tetrazolium, NOX, NADPH oxidase, ROS, reactive oxygen species, SOD, superoxide dismutase, YPD, yeast extract peptone dextrose

## Abstract

Across eukaryotes, Rho GTPases such as Rac and Cdc42 play important roles in establishing cell polarity, which is a key feature of cell growth. In mammals and filamentous fungi, Rac targets large protein complexes containing NADPH oxidases (NOX) that produce reactive oxygen species (ROS). In comparison, Rho GTPases of unicellular eukaryotes were believed to signal cell polarity without ROS, and it was unclear whether Rho GTPases were required for ROS production in these organisms. We document here the first example of Rho GTPase–mediated post-transcriptional control of ROS in a unicellular microbe. Specifically, Cdc42 is required for ROS production by the NOX Fre8 of the opportunistic fungal pathogen *Candida albicans*. During morphogenesis to a hyphal form, a filamentous growth state, *C. albicans FRE8* mRNA is induced, which leads to a burst in ROS. Fre8-ROS is also induced during morphogenesis when *FRE8* is driven by an ectopic promoter; hence, Fre8 ROS production is in addition controlled at the post-transcriptional level. Using fluorescently tagged Fre8, we observe that the majority of the protein is associated with the vacuolar system. Interestingly, much of Fre8 in the vacuolar system appears inactive, and Fre8-induced ROS is only produced at sites near the hyphal tip, where Cdc42 is also localized during morphogenesis. We observe that Cdc42 is necessary to activate Fre8-mediated ROS production during morphogenesis. Cdc42 regulation of Fre8 occurs without the large NOX protein complexes typical of higher eukaryotes and therefore represents a novel form of ROS control by Rho GTPases.

Reactive oxygen species (ROS) including superoxide anion radical and hydrogen peroxide (H_2_O_2_) have dual faces in biology. While ROS can cause severe oxidative damage to biomolecules, they can also be exploited as signaling molecules, particularly ROS originating from NADPH oxidase (NOX) enzymes (1, 2). In these large membrane proteins, electrons from NADPH pass through a flavin and heme to reduce molecular oxygen to superoxide. The superoxide is then converted to H_2_O_2_, either by spontaneous dismutation or by a superoxide dismutase (SOD) enzyme. The diffusible H_2_O_2_ then acts as a signaling molecule to elicit a wide array of downstream cellular events (1, 2, 3, 4).

Because of the potential toxicity of ROS, NOX enzymes are tightly regulated, and much of this regulation occurs at the post-translational level of enzyme activation. In plants, insects, and a subset of mammalian and higher fungi, NOX enzyme activation occurs through calcium fluxes and EF hands within domains of the NOX polypeptide (5). In mammals and higher fungi, activation can also occur through assembly of large NOX enzyme complexes with a Rho GTPase and multiple accessory proteins. The prototype is phagocytic NOX2 of the immune system that is activated by the Rac Rho GTPase. When stimulated, Rac activates NOX through physical interactions with a regulatory subunit p67. Once Rac engages p67, the complex activates electron flow through NOX from NADPH to oxygen. The NOX complex also includes an adaptor protein p22 and accessory components p47 and p40 (5, 6). In the fungal kingdom, Rac activation of NOX has been well studied in filamentous or multicellular fruiting body forming fungi. Analogous to mammals, Rac activates NOX through a regulator NoxR, equivalent to animal p67. Here, we shall refer to this essential regulator as NoxR/p67. Filamentous fungi also use NOX adaptors Bem (equivalent to animal p40 and/or p47) and NoxD (p22) (7, 8). ROS from Rac-activated fungal NOX is known to signal polarized cell growth across diverse classes of filamentous fungi (8, 9, 10, 11, 12, 13, 14).

In unicellular yeasts, polarized growth is also spatially and temporally regulated by Rho GTPases, and the best studied example is Cdc42 (15). This Rho GTPase is essential for bud formation for cell division in *Saccharomyces cerevisiae* (16) and in the opportunistic fungal pathogen *Candida albicans* (17). Cdc42 is in addition used for cell polarization during mating in *S. cerevisiae* (18), and Cdc42 is essential for hyphal morphogenesis of *C. albicans* (19, 20). *C. albicans* also expresses a Rac1 Rho GTPase that participates in a distinct cell polarization pathway associated with substrate invasion (21). As with metazoans, yeast Rho GTPases control cell polarity through numerous effector proteins and kinase signaling pathways (15, 16, 22). However, NOX is not a known effector of Rho GTPases in yeasts. There has been no documentation of NOX activation by Rho GTPases in any unicellular organism. Yeast genomes lack the NoxR/p67 regulator that is essential for Rac activation of NOX in higher fungi and mammals and are also missing the adaptor protein NoxD/p22. Until recently, yeasts were believed to lack NOX enzymes (8, 23, 24).

The first NOX identified for yeasts was *S. cerevisiae* Yno1 that acts in the endoplasmic reticulum for actin polymerization (25). In 2017, we identified a NOX known as Fre8 (or Cfl11) in *C. albicans* that functions during hyphal morphogenesis (26). *C. albicans* is polymorphic and can grow either as a yeast form, similar to *S. cerevisiae*, or as hyphal filaments that are important for host cell invasion (27). Fre8 specifically produces ROS in hyphal cells at the site of polarized growth. In laboratory cultures, Fre8 ROS is not essential for morphogenesis but contributes to sustaining late stage filamentation. In rodent models of infection, *fre8* deletion mutants form attenuated biofilms on implanted devices and exhibit shortened hyphae in disseminated candidiasis (26). *FRE8* is transcriptionally induced in hyphal cells coincident with the ROS burst of morphogenesis (26). To date, there have been no reports of post-transcriptional control of Fre8 ROS. There has also been no evidence for post-translational regulation of ROS by Fre8, particularly since *C. albicans* lacks the NOX regulatory subunits that are essential targets of Rho GTPases in higher organisms.

Here, we provide evidence for the first time that a NOX from a unicellular yeast is indeed controlled at the post-transcriptional level, and like higher eukaryotes, this regulation involves a Rho GTPase. In *C*. *albicans*, Cdc42 is required for the ROS burst of hyphal morphogenesis produced by Fre8 NOX. Surprisingly, this activation of NOX occurs in the absence of the NOX accessory factors used by higher fungi and animals. *C. albicans* Cdc42 control of Fre8-ROS may represent the most simple form of Rho GTPase control of ROS for polarized growth.

## Results and discussion

### Analysis of Fre8 localization

One method of post-transcriptional control of NOX in higher fungi involves protein localization. Specifically, in the filamentous fungi *Neurospora crassa*, most of NoxA is held in an inactive state in the vacuolar system, whereas only NoxA at the hyphal tip and plasma membrane is active for ROS production (7). To test whether the same may apply to *C. albicans* Fre8, we analyzed a fusion of Fre8 to dTomato (Fre8-dTom) for its enzymatic activity *versus* localization in cells undergoing hyphal morphogenesis.

**Fig. 1:**
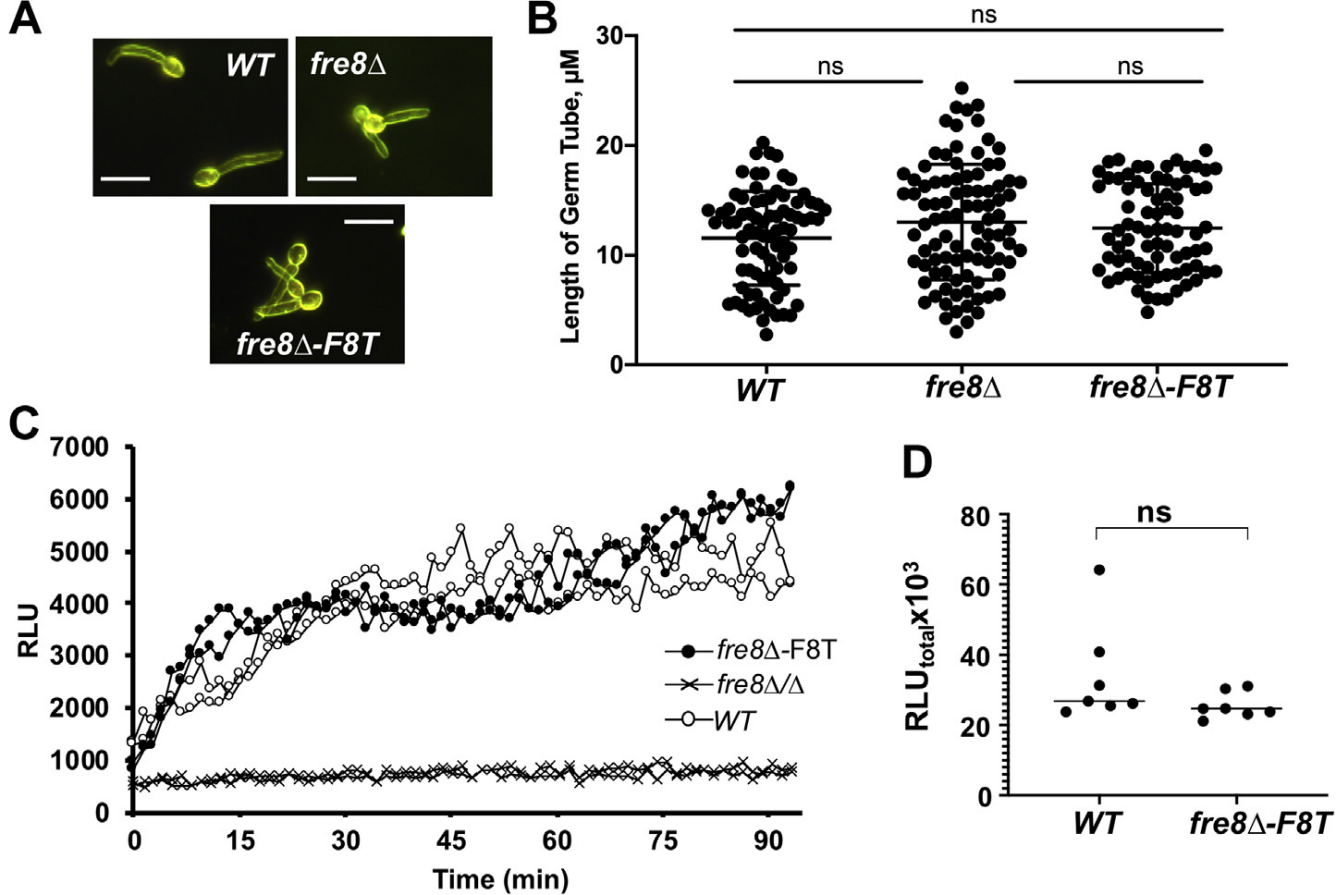
**A Fre8-dTomato (Fre8-dTom) fusion is active in ROS production.** The indicated strains were stimulated to form germ tubes by incubation for 1 h in IMDM. *A*, cells were photographed by dark field microscopy and (*B*) germ tube length measured in approximately 200 cells from duplicate cultures of WT, *fre8Δ*, and *fre8-F8T* strains. There is no statistically significant difference as determined by one-way ANOVA. The bar represents 10 microns. *C*, two independent cultures of each strain were subjected to luminol chemiluminescence measurements of ROS as described in Experimental procedures section. Results were recorded as relative luminescence units (RLUs) and plotted in intervals of minutes. The *fre8Δ/Δ* strain shows undetected ROS in this assay and gives signals equivalent to background luminescence. *D*, total luminol luminescence over 90 min was calculated as described in the Experimental procedures section. Data points represent six independent cultures from three experimental trials. The bar represents mean; ns indicates no statistically significant difference as determined by two-tailed *t* test. The following strains were utilized: WT, SC5314, *fre8Δ/Δ*, CA-JG211; *fre8Δ*-F8T, CA-JG211 expressing Fre8-dTom. IMDM, Iscove's modified Dulbecco's medium; ROS, reactive oxygen species.

**Fig. 2:**
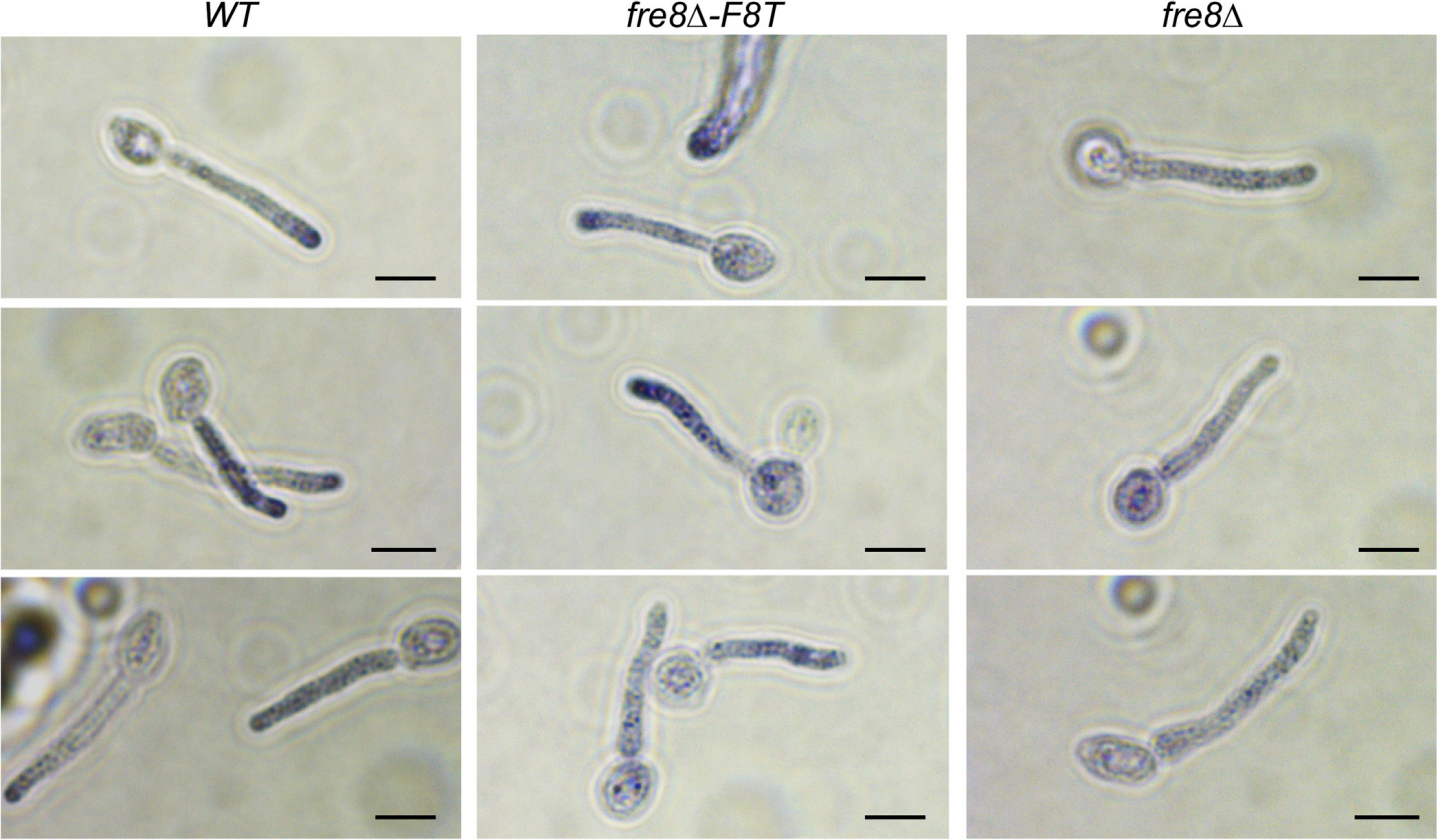
**NBT staining of cells expressing Fre8-dTom.** The indicated strains were stimulated to form germ tubes and stained with NBT as described in the Experimental procedures section. Polarized staining toward the hyphal tip is seen in WT and Fre8-dTom–expressing strains, whereas *fre8Δ/Δ* strains show light punctate staining throughout the cell. About 70% of WT (26 of 37 individual cells) and 50% of the Fre8-dTom–expressing germ tubes (30 of 59 cells) across two independent cultures showed polarized staining with NBT. Similar trends were observed in three experimental trials, and strains are as described for Figure 1. The bar represents 5 microns. Fre8-dTom, Fre8 to dTomato; NBT, nitroblue tetrazolium.

Fre8-dependent ROS in hyphal cells can be readily monitored using luminol chemiluminescence (26). In the experiment mentioned in Figure 1, *C. albicans* was induced to form short hyphae or germ tubes by incubating for 1 h in Iscove's modified Dulbecco's medium (IMDM), a medium that promotes morphogenesis through high amino acids (26). Consistent with previous studies (26), *fre8Δ/Δ* cells show no hyphal defect under these laboratory conditions, and expression of Fre8-dTom also did not impact germ tube formation or hyphal length (Fig. 1, *A* and *B*). By luminol chemiluminescence, *fre8Δ/Δ* germ tubes show no ROS signal above background (Fig. 1*C*). Expression of Fre8-dTom in *fre8Δ/Δ* cells restored ROS production to near WT levels (Fig. 1, *C* and *D*). To examine localization of ROS production, we employed nitroblue tetrazolium (NBT) staining. The reduction of NBT by superoxide either inside or outside the cell produces blue formazan that can be visualized by light microscopy. With WT cells, many germ tubes show polarized NBT staining toward the hyphal tip, in contrast to the uniform light background staining of *fre8Δ/Δ* mutants (Fig. 2). Intensified NBT staining toward the hyphal tip was also seen in a number of cells expressing Fre8-dTom (Fig. 2), similar to vesicular NBT staining reported for filamentous fungal NOX at sites of polarized growth (13, 28). Thus, Fre8-dTom is active for ROS production, and the activity concentrates toward the hyphal tip.

**Fig. 3:**
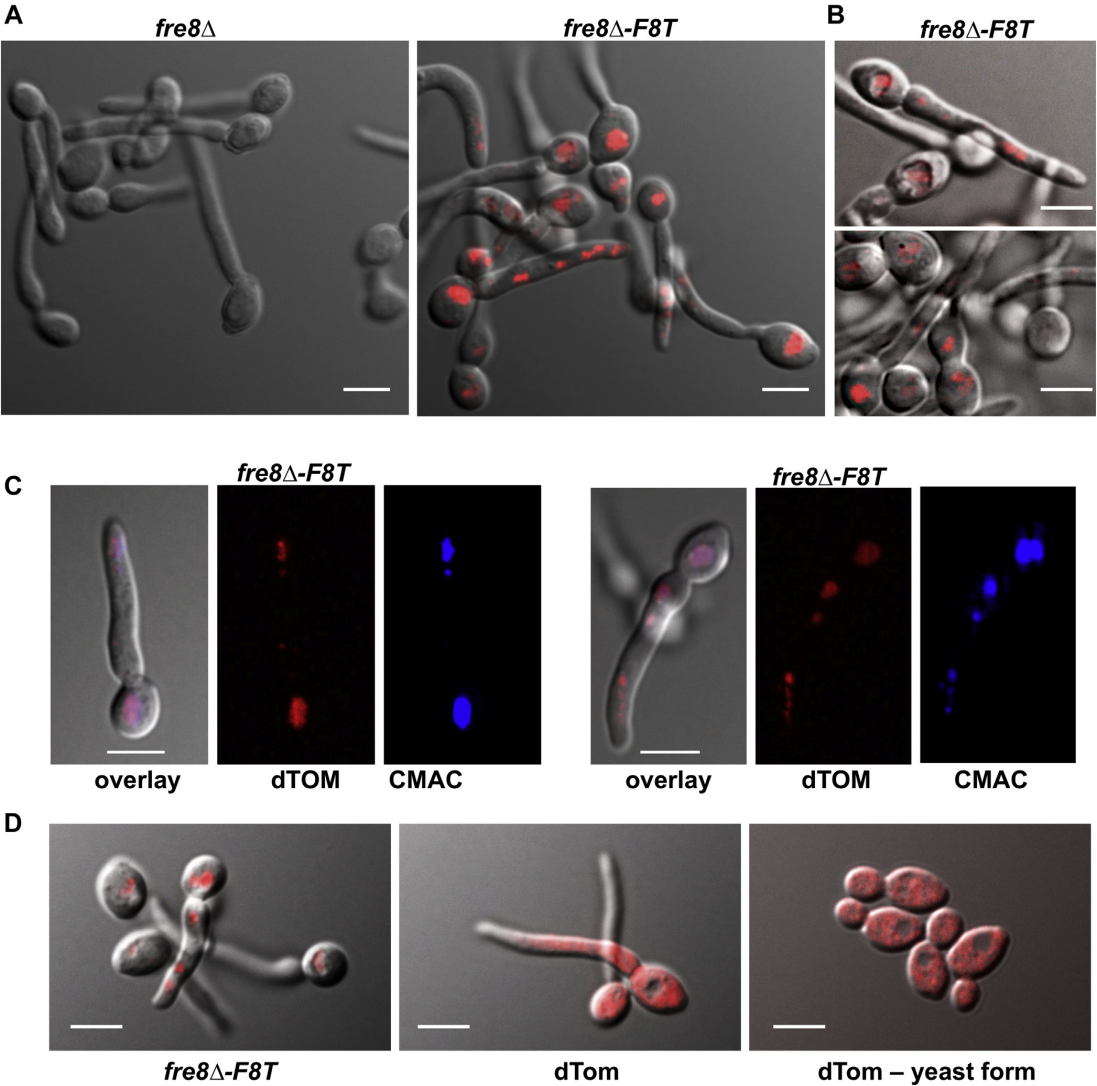
**Fre8-dTom is in the vacuolar system.** The indicated strains were induced to form hyphae in IMDM (*A*, *C*, *D*, *left* and *middle panels*) or in serum (*B*). *D*, *right panel*, cells were not induced. Cells were analyzed by fluorescence and differential interference contrast microscopy as described in the Experimental procedures section, and overlay images are shown. *C*, cells were exposed to the vacuolar dye CMAC in the last 30 min of hyphal formation as described in the Experimental procedures section. Fluorescent microscopy studies of Fre8-dTom localization with IMDM and serum-induced germ tubes are representative of four and two experimental trials, respectively. Experiments with CMAC staining (*C*) are representative of three cultures over two experimental trials. The following strains were utilized: *fre8Δ* and *fre8Δ-F8T* as described in Figure 1; dTom is SC5314-expressing isolated dTomato. The bar represents 5 microns. CMAC, 7-amino-4-chloromethylcoumarin; Fre8-dTom, Fre8 to dTomato; IMDM, Iscove's modified Dulbecco's medium.

Based on the pattern of NBT staining ((26) and Fig. 2), we predicted a Fre8-dTom localization at the hyphal tip. However, much of Fre8-dTom is in the main vacuole of the mother cell as defined by indentations with light microscopy, as well as in compartments within the germ tube, whether cells were induced for hyphal growth with IMDM (Fig. 3*A*) or serum (Fig. 3*B*). To further define Fre8-dTom localization, we employed the vacuolar dye 7-amino-4-chloromethylcoumarin (CMAC) (29), and Figure 3*C* shows that Fre8-dTOM colocalizes with CMAC. Compared with the vacuolar network localization of Fre8-dTOM, free dTom exhibits diffuse cytoplasmic staining excluded from the vacuole in both germ tubes and budding yeast-form cells (Fig. 3*D*).

**Fig. 4:**
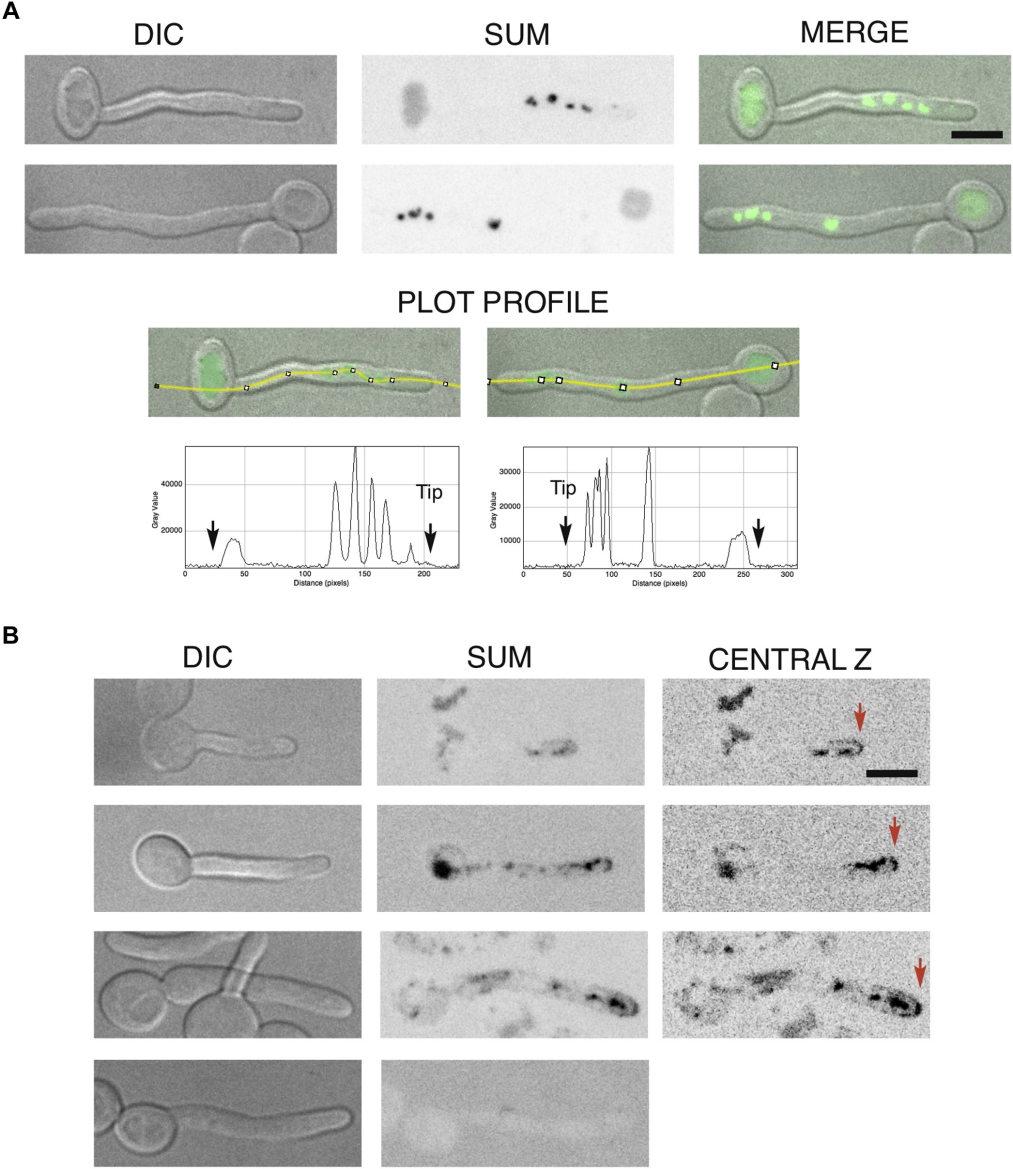
**Fre8 is preferentially localized to the hyphal apex.** Cells expressing Fre8-GFP from the endogenous locus were induced to form hyphae in the presence of 50% serum for 90 min (*A*) or 70 min (*B*) and analyzed by confocal microscopy as described in the Experimental procedures section. The bar represents 5 microns. *A*, Fre8 is more concentrated at the apex. Differential interference contrast (DIC), sum projections, and merge images of cells expressing Fre8-GFP (PY6053) are shown, together with a plot intensity profile in which *arrows* indicate the front (tip) and back of the filamentous cells. *B*, Fre8 is associated with the cortex at the filament tip. DIC, sum projections, and central z-section of cells expressing Fre8-GFP (PY6053) are shown (*top three rows*). DIC and sum projections of a WT cell (PY4860) are shown as control in the *bottom row*.

Vacuolar localization was also recently reported for a Fre8-GFP fusion expressed ectopically under the *ACT1* promoter (30). In Figure 3, Fre8-dTom is ectopically expressed under *ENO1*, and we therefore addressed whether the protein may be mislocalized because of overexpression. As seen in Figure 4*A*, cells expressing *FRE8*-GFP as a sole copy from the endogenous promoter at the *FRE8* locus exhibit a similar distribution as cells expressing *FRE8-dTom* ectopically, following 90 min of serum induction. Fre8 was observed in the vacuole of the mother cell, as well as in the filament, in a compartment likely to be fragmented vacuoles. In this compartment, the concentration of Fre8 is increased compared with that of the mother cell vacuole (see *line plot profiles*, *bottom panel*; Fig. 4*A*). Furthermore, it is noteworthy that at earlier serum induction times, that is, 60 to 70 min, we also observed cells with additional fluorescence signal associated with the cortical apex (Fig. 4*B*). This low cortical signal was not seen when *FRE8* was expressed from its endogenous promoter at later times, suggesting that a fraction of Fre8 may transiently associate with the plasma membrane. As this cortical signal was also not seen when *FRE8* was expressed from the *ENO1* promoter, transient induction and expression level may be critical.

Although Fre8 can be seen mainly throughout the vacuolar network (Figs. 3 and 4), Fre8-derived ROS is focused toward the hyphal apex (Fig. 2), and it is probable that much of the protein in the vacuolar network is inactive, similar to fungal NoxA of *N. crassa* (7). By fluorescence microscopy, we observed Fre8 in compartments close to the hyphal tip (Figs. 3 and 4), and it is likely that these are responsible for the ROS generated, as has been reported for vesicular NOX of certain filamentous fungi (13, 28). We originally predicted a plasma membrane localization for Fre8, since an extracellular SOD5 can react with superoxide derived from Fre8 (26), and it is indeed possible that a small fraction of Fre8 may transiently associate with the apical cortex (Fig. 4*B*). In any case, whether in apical compartments or cortex, only Fre8 in the hyphal apex would be active for ROS production, indicative of post-translational activation.

### ROS production by ectopically expressed *FRE8*

**Fig. 5:**
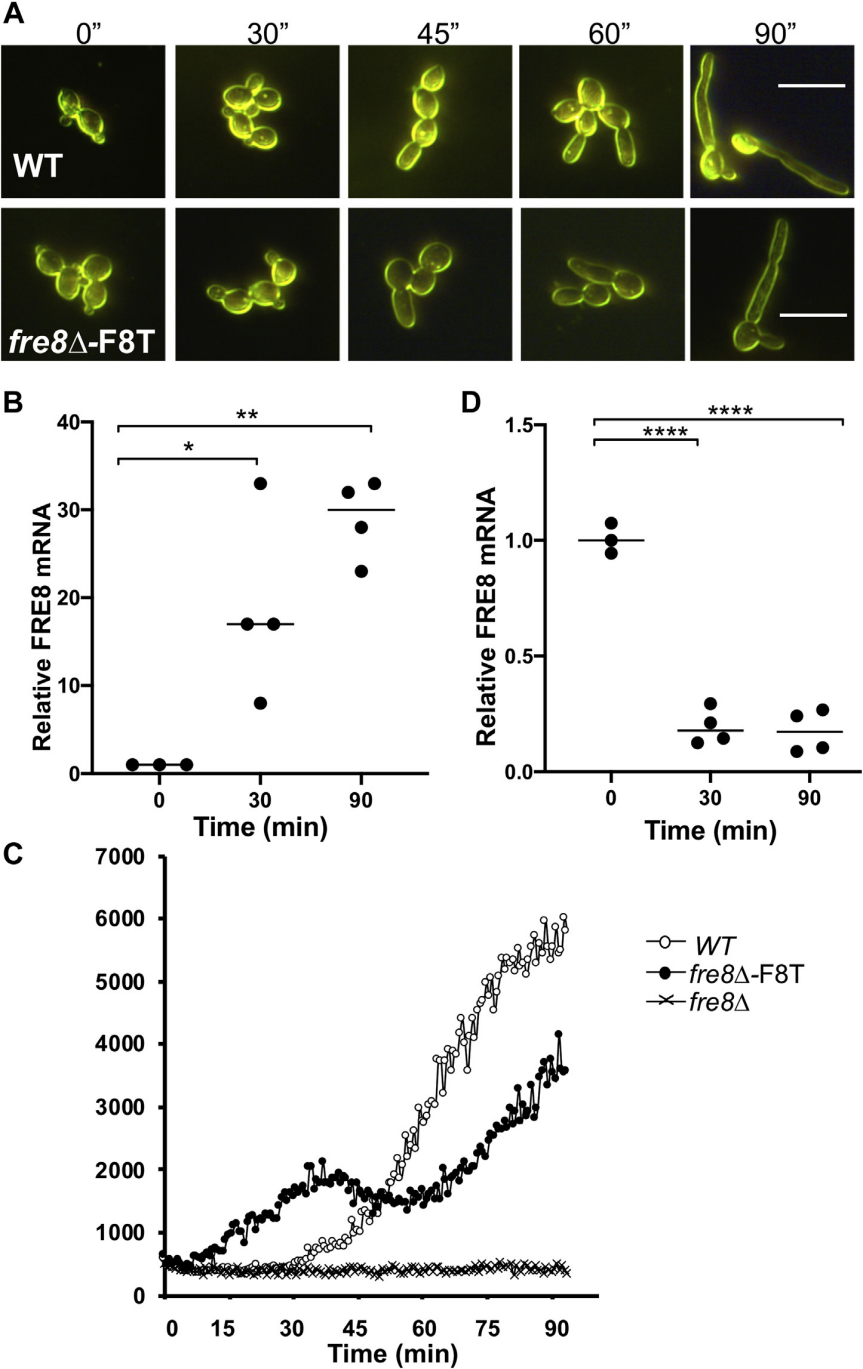
**Temporal pattern of ROS production from ectopically expressed *FRE8*.** The indicated strains as described for Figure 1 in yeast-form budding state were induced to form extended germ tubes in the luminol assay for ROS as described in the Experimental procedures section. *A*, dark field microscopy images were taken at the indicated time points. The bars represent 10 microns. *B* and *D*, RNA from WT (*B*) and the expressing *FRE8-dTom* under *ENO1* (*D*) was subjected to quantitative RT-PCR analysis of *FRE8* expression. Results shown are fold change in *FRE8* mRNA over that of control budding cells at *t* = 0. Data points are from three to four independent cultures and two experimental trials. ∗*p* = 0.018; ∗∗*p* = 0.0014; and ∗∗∗∗*p* = 0.0001 as determined by one-way ANOVA. *C*, luminol chemiluminescence measurements of ROS were carried out as for Figure 1*A*. Results are representative of four experimental trials. Fre8-dTom, Fre8 to dTomato; ROS, reactive oxygen species.

*FRE8* is well known to be regulated at the transcriptional level. *FRE8* mRNA is of very low abundance in budding yeast-form cells but is strongly induced during hyphal morphogenesis (26, 31). We can temporally monitor this induction of *FRE8* in real time using luminol chemiluminescence. In the experiment mentioned in Figure 5, yeast cells were induced to form germ tubes during the chemiluminescence assay by shifting the temperature to 37 ^o^C and by including IMDM in the reaction mix. After 30 to 45 min, cells began to form visible germ tubes that progressively lengthen throughout the course of the reaction (Fig. 5*A*). In WT cells, *FRE8* mRNA (Fig. 5*B*) and Fre8-dependent ROS (Fig. 5*C*) are induced concomitant with this morphogenesis.

We also examined the time course of ROS production when *FRE8* is ectopically expressed from *ENO1* using our Fre8-dTom fusion. Unlike WT cells that produce no ROS in yeast-form cells during the first 30 min, the yeast-form cells expressing Fre8-dTom from *ENO1* emit ROS during this phase (Fig. 5*C*). This finding is consistent with our previous results showing Fre8-ROS production in yeast-form cells that ectopically express *FRE8* (26). As cells transition to germ tubes, *ENO1*-driven *FRE8* mRNA levels vary opposite to that observed with endogenous *FRE8*, with mRNA levels of *FRE8-dTom* dropping ≈10-fold (Fig. 5*D*). Surprisingly, ROS production from *ENO1*-driven Fre8-dTom did not follow this reduction in mRNA level, as a second wave of ROS production initiated at 60 min as morphogenesis proceeded (Fig. 5*C*). This post-transcriptional regulation of *FRE8* is consistent with our studies of Fig. 2, Fig. 3, Fig. 4 indicating control of Fre8 activity at the post-translational or protein localization level.

Collectively, our studies indicate that Fre8 production of ROS proceeds by two steps during morphogenesis. First, *FRE8* mRNA is induced 10- to 100-fold in cells induced to form hyphae as part of the transcriptional reprogramming for morphogenesis (26, 31). Second, independent of transcription, Fre8 is activated for ROS production (Fig. 5), specifically at the hyphal tip (Fig. 2), while much of Fre8 exists in the vacuolar network in an apparent inactive state (Figs. 3 and 4). Such spatial control of Fre8 activity is highly reminiscent of what has been reported for NOX of *N. crassa* and *Aspergillus fumigatus.* In these filamentous fungi, NOX is only active at the site of polarized growth when the enzyme engages the Rho GTPase RacA and NoxR/p67 regulator (7, 13). Mammalian NOX2 has also been reported to exist in an inactive holding state in lysosomes and becomes active when the enzyme contacts Rac and NoxR/p67 in the phagolysosome (32). Is *C. albicans* Fre8 likewise regulated by a Rho GTPase?

### Rho GTPases and regulation of *C. albicans* Fre8-ROS

**Fig. 6:**
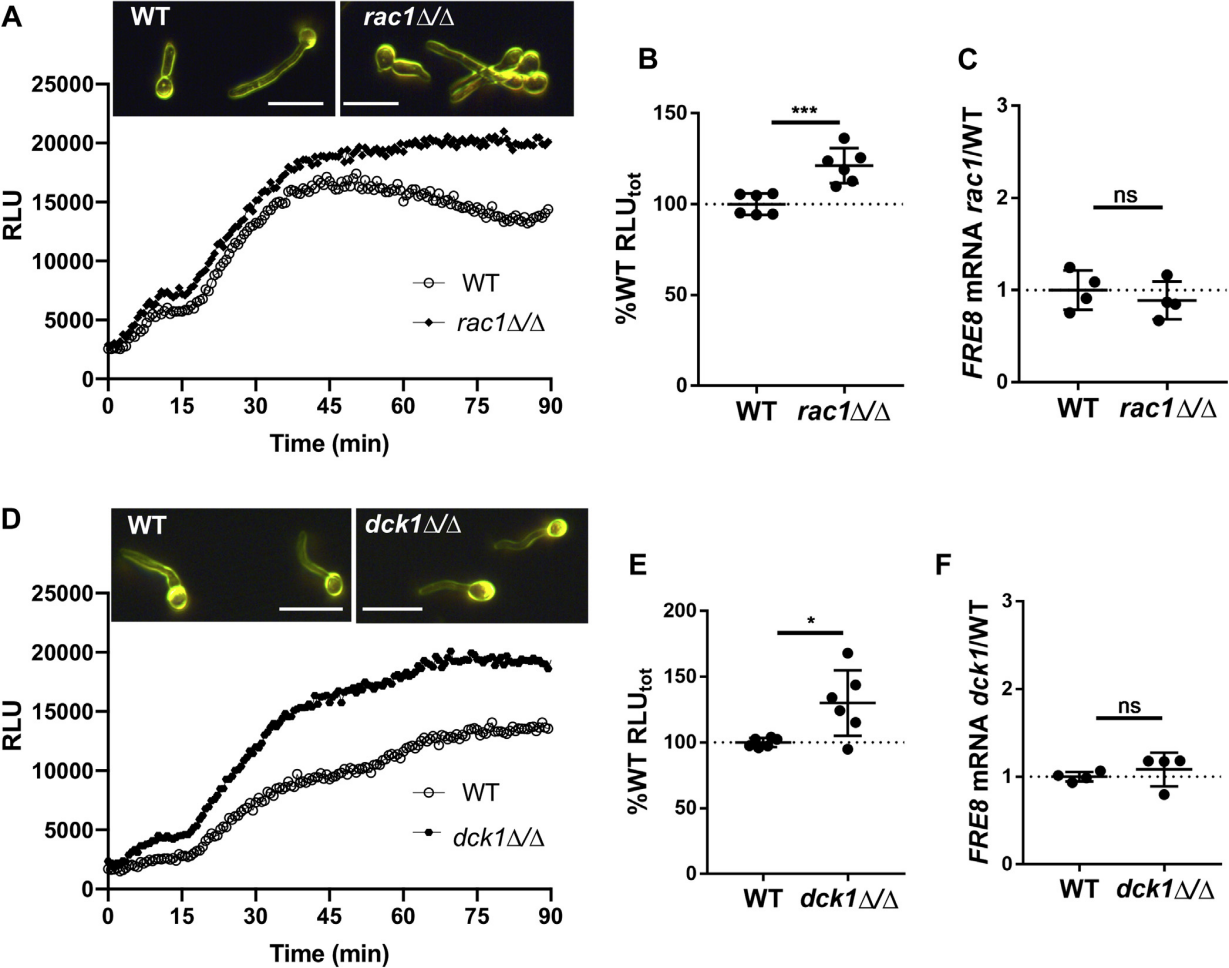
**The Rho GTPase Rac1 and Fre8 ROS**. The indicated strains were induced to form germ tubes by 1 h incubation in IMDM. *A* and *D*, cells were subjected to dark field microscopy (*top*) and for assays of Fre8 ROS by luminol chemiluminescence (*bottom*) as for Figure 1, *A* and *C*. The bars represent 10 microns. *B* and *E*, ROS production as a function of total luminol luminescence was determined as for Figure 1*D* and plotted as percent change over WT luminescence = 100%. Values are from six cultures over three independent trials. *C* and *F*, *FRE8* mRNA was analyzed by quantitative RT-PCR and plotted as fold change over WT levels. Results are from four cultures over two independent trials. The bar represents mean; error bars represent SD. ∗*p* = 0.0154; ∗∗∗*p* = 0.001 as determined by two-tailed *t* test. The following strains were utilized: (*A*–*C*) WT, BWP17; *rac1Δ*/*Δ*, PY189; (*D*–*F*) WT, SN250; *dck1Δ*/*Δ*, SN250:*dck1*Δ/Δ. IMDM, Iscove's modified Dulbecco's medium; ns, not significant; ROS, reactive oxygen species.

Although there is no NoxR/p67 in the genomes of yeasts, *C. albicans* does express a Rac1 Rho GTPase that is needed for invasion of solid substrates (21). We therefore tested the possible role of Rac1 in regulation of Fre8 ROS. *C. albicans rac1Δ/Δ* mutants are capable of forming germ tubes in liquid IMDM (Fig. 6*A*, *top*), consistent with other studies in liquid cultures (21). Using the luminol chemiluminescence assay, we observed no loss in Fre8 ROS in the *rac1Δ/Δ* mutant (Fig. 6*A*, *bottom*), and if anything, ROS was somewhat elevated in this mutant across numerous experimental trials (Fig. 6*B*). This increase in Fre8 ROS occurs independent of any changes in *FRE8* mRNA levels (Fig. 6*C*).

Rac1 in *C. albicans* is activated by the guanine nucleotide exchange factor Dck1p (33), hence, we examined Fre8-ROS in the *dck1Δ/Δ* strain. As seen in Figure 6, *D*–*F*, similar results were obtained with *dck1Δ/Δ* mutants, where Fre8-ROS is somewhat elevated (Fig. 6, *D* and *E*), without changes in *FRE8* mRNA levels (Fig. 6*F*). Therefore, unlike filamentous fungi and animals, *C. albicans* Fre8 is not dependent on Rac1 for activity and, if anything, may be negatively impacted by this Rho GTPase activity.

**Fig. 7:**
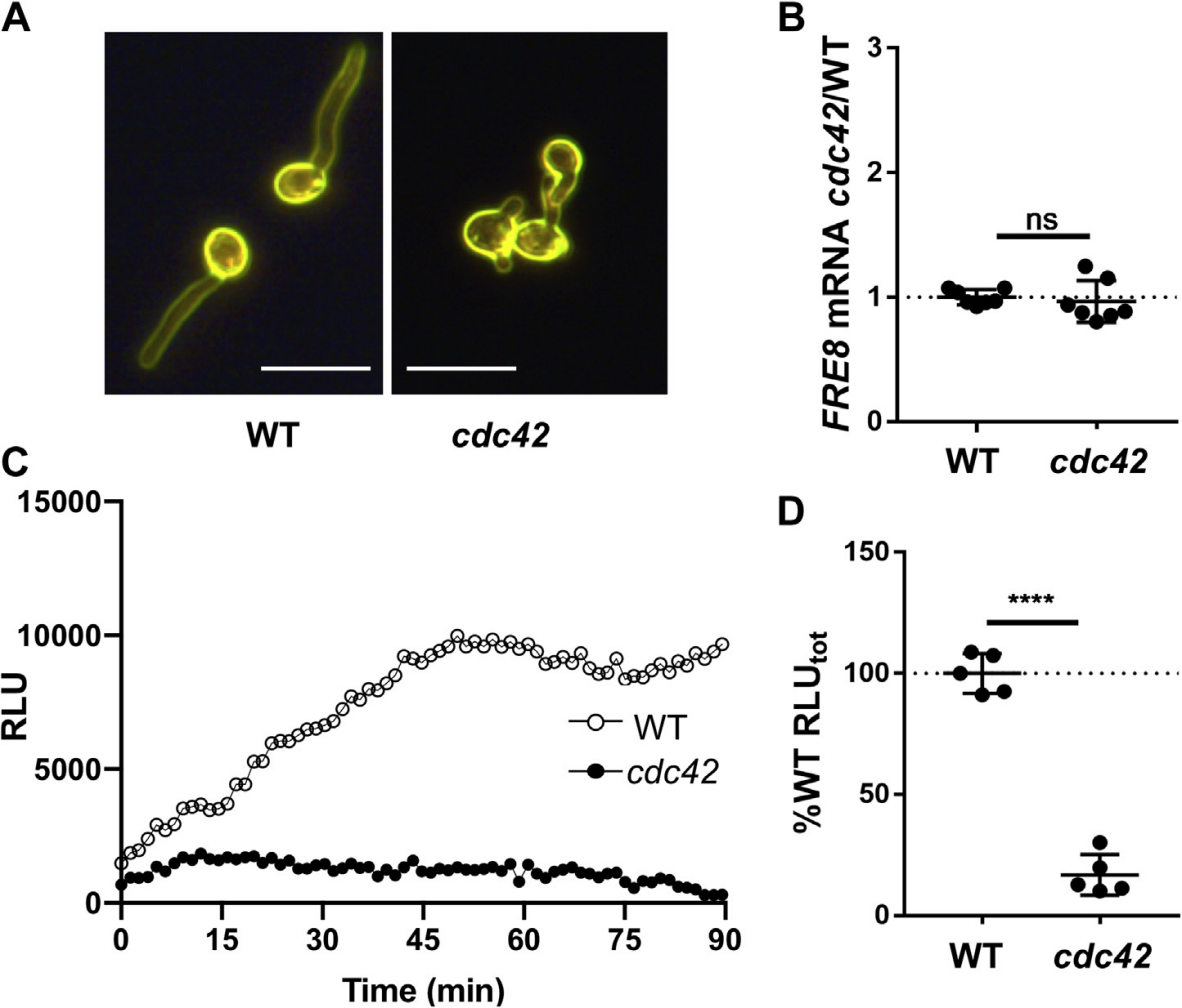
**Fre8-dependent ROS is greatly inhibited by loss of Cdc42.** The indicated strains were induced to form germ tubes in the presence of methionine to repress *MET2*-driven *CDC42* in the *cdc42* mutant. *A*, cells imaged by dark field microscopy show short germ tubes in the *cdc42* mutant. The bars represent 10 microns. *B*, *FRE8* mRNA levels were analyzed in seven independent cultures across three experimental trials as for Figure 6*C*. *C* and *D*, ROS was monitored by luminol chemiluminescence as for Figure 6, *A* and *B*. *D*, results are from five independent cultures from three experimental trials. The bar represents mean; error bars represent SD. ∗∗∗∗*p* < 0.0001 as determined by two-tailed *t* test. The following strains were utilized: WT, BWP17; *cdc42*, PY47. ns, not significant; ROS, reactive oxygen species.

**Fig. 8:**
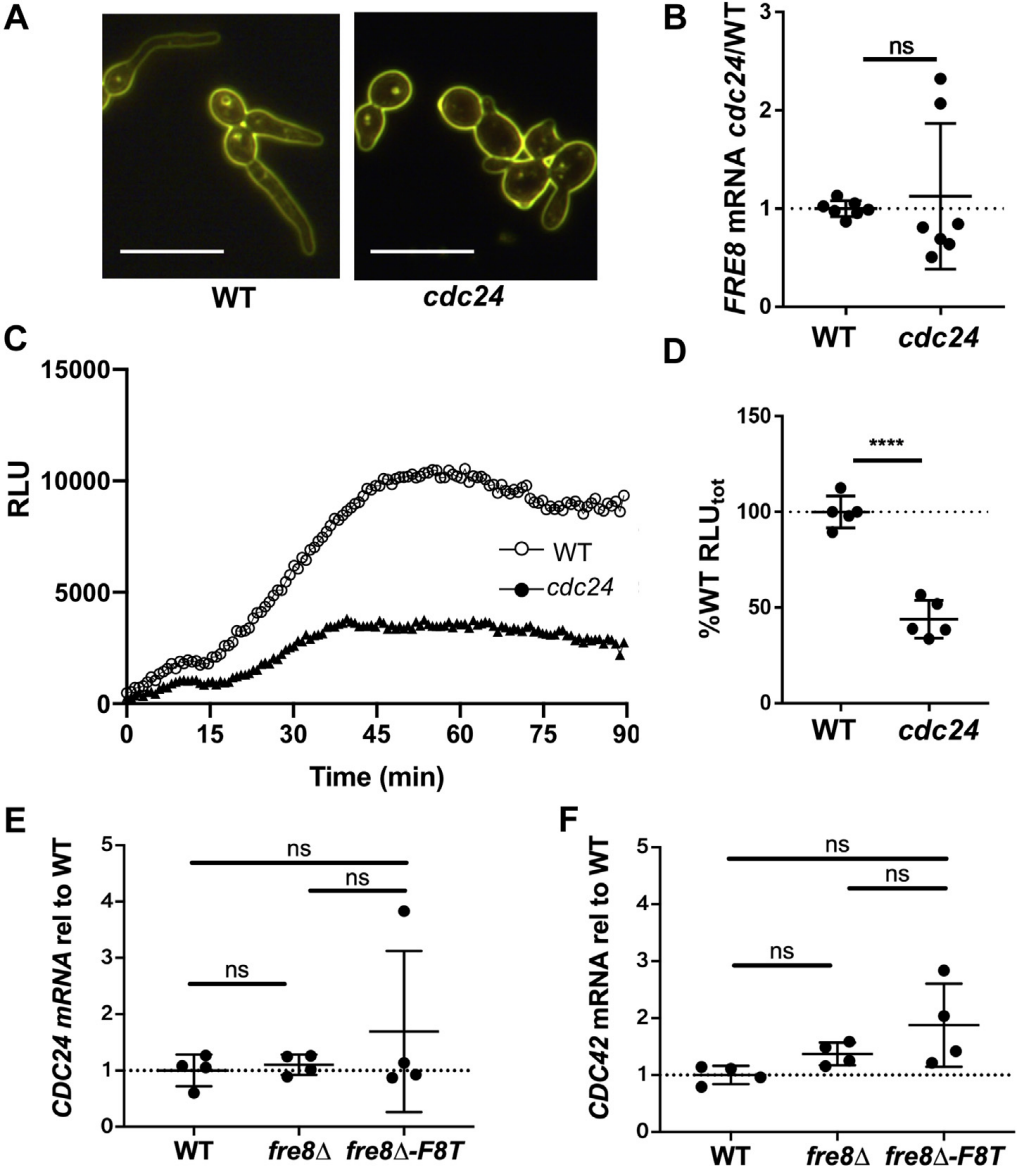
**Fre8 dependent ROS and the Cdc24 GEF for Cdc42.***A*–*D*, germ tubes of the designated strains were formed under conditions to repress *MET2*-driven *CDC24* as described for *CDC42* for Figure 7. Cells were imaged (*A*), *FRE8* mRNA analyzed (*B*), and ROS measured by luminol chemiluminescence (*C* and *D*) as for Figure 6. The bars in part A represent 10 microns. Data represent seven (*B*) or five cultures (*D*) in three experimental trials. The bar represents mean; error bars represent SD. ∗∗∗∗*p* < 0.0001 as determined by two-tailed *t* test. The following strains were utilized: WT, BWP17; *cdc24*, PY101. *E* and *F*, *CDC24* and *CDC42* mRNA was quantitated by quantitative RT-PCR in the indicated strains induced to form germ tubes in IMDM. Data represent four independent cultures over two trials. Differences were not significant as determined by one-way ANOVA. GEF, guanine nucleotide exchange factor; IMDM, Iscove's modified Dulbecco's medium; ns, not significant; ROS, reactive oxygen species.

A second Rho GTPase in *C. albicans* is Cdc42, and we tested whether Fre8 ROS is regulated by this GTPase. *C. albicans* Cdc42 is essential for viability (17), and overexpression of a constitutively active Cdc42 allele is deleterious for growth (20); hence, we employed a *Δcdc42/*pMet*CDC42* strain expressing *MET3*-repressible *CDC42* that is viable in media lacking methionine (19, 34). In the experiment mentioned in Figure 7, cells were initially cultured in media lacking methionine, then *CDC42* was repressed by adding methionine in the final 2 h of yeast-form growth and during the 1 h induction of germ tubes. These cells with repressed *CDC42* produced short germ tubes (Fig. 7*A*), consistent with previous studies (19, 20, 34). In spite of this stunted morphogenesis, *FRE8* mRNA was induced, and there was no significant difference in *FRE8* expression in WT *versus Δcdc42/*pMet*CDC42* cells (Fig. 7*B*). However, a dramatic impact on Fre8-ROS was observed, as ROS levels were reduced nearly 10-fold in *Δcdc42/*pMet*CDC42* cells (Fig. 7, *C* and *D*). This loss in ROS is not because of the reduced hyphal length. As mentioned previously, Fre8 can produce ROS in the absence of hyphae, specifically in yeast-form cells ectopically expressing *FRE8* ((26) and Fig. 5*C*). To examine the requirement for Cdc42 activity further, we tested the importance of Cdc24, the unique guanine nucleotide exchange factor for Cdc42 required for viability, using a similar *MET3*-repressible strain, *Δcdc24/*pMet*CDC24* (19). As seen in Figure 8*A*, cells with repressed *CDC24* also have short germ tubes consistent with previous findings (19, 34). As with Cdc42 (Fig. 7, *B*–*D*), repression of *CDC24* expression resulted in a reduction of Fre8-ROS (Fig. 8, *C* and *D*) without significant changes in *FRE8* mRNA (Fig. 8*B*).

**Fig. 9:**
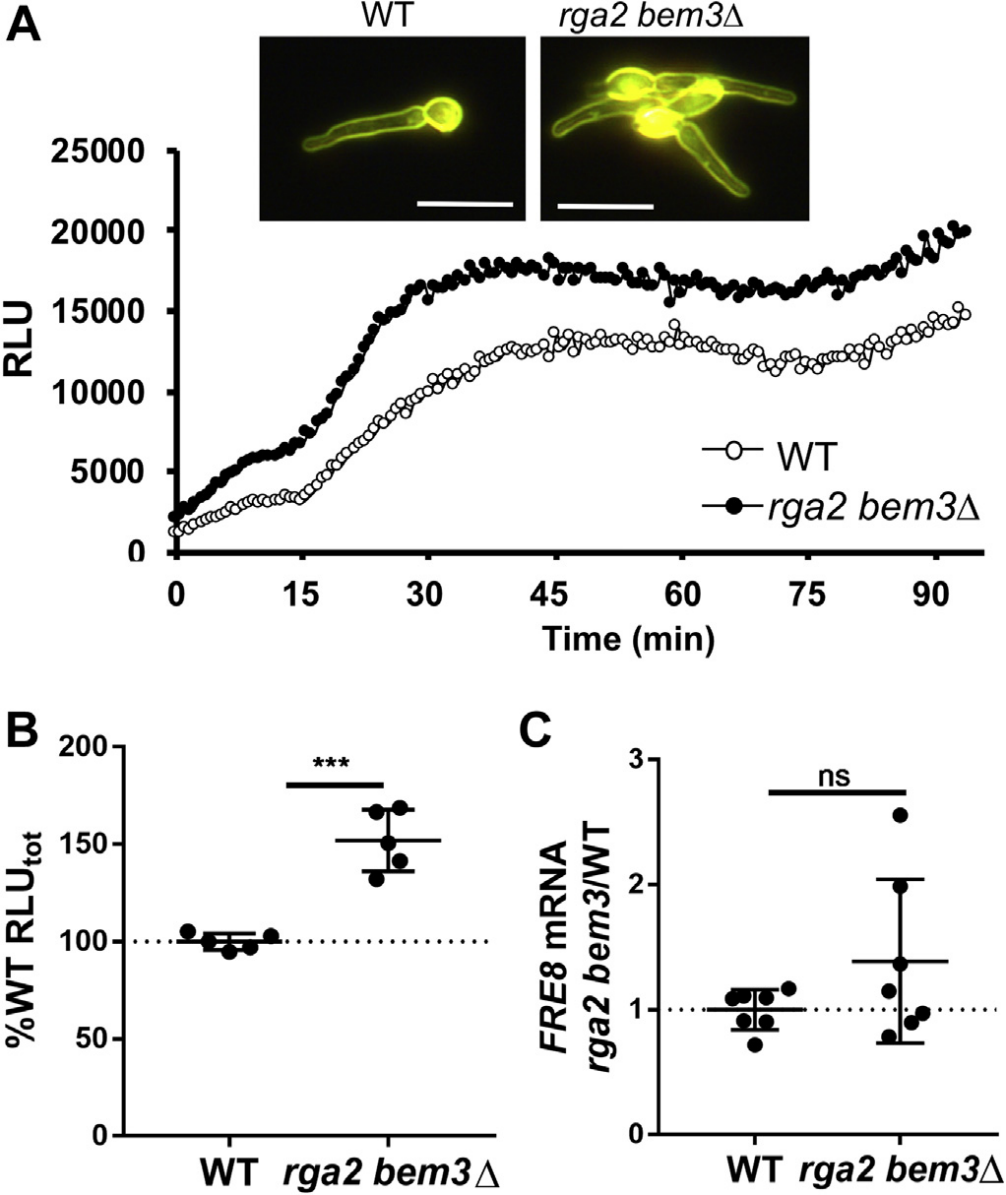
**ROS enhancement with the GAP for Cdc42.** The indicated strains were induced to form germ tubes and subjected to dark field microscopy (*A, top*) and for assays of Fre8 ROS by luminol chemiluminescence (*A, bottom* and *B*) and for *FRE8* mRNA (*C*) as described for Figure 6. The bar in part A represents 10 microns. Data represent five (*B*) or seven cultures (*C*) from three experimental trials. The bar represents mean; error bars represent SD. ∗∗∗*p* = 0.0001 as determined by two-tailed *t* test. The following strains were utilized: WT, BWP17; *rga2 bem3Δ*, 615. GAP, GTPase-activating protein; ns, not significant; ROS, reactive oxygen species.

*C. albicans* Cdc42 activity is negatively regulated by the GTPase-activating proteins Rga2 and Bem3 (35, 36). A double *rga2 bem3* null strain is viable (35, 36), and there was no obvious impact on germ tube formation in IMDM (Fig. 9*A*, *top*). We observed that Fre8-ROS was reproducibly increased in the *rga2 bem3Δ/Δ* strain (Fig. 9*A*, *bottom*, and *B*), without significant changes in *FRE8* mRNA (Fig. 9*C*), consistent with these GTPase-activating proteins negatively regulating Cdc42.

Together, the findings of Fig. 7, Fig. 8, Fig. 9 support the notion that Cdc42 positively regulates Fre8-ROS. The effect of Cdc42 occurs in the absence of changes in *FRE8* mRNA, and the converse is true: there are no statistically significant changes in *CDC42* and *CDC24* mRNA as a function of *FRE8* expression (Fig. 8, *E* and *F*). In contrast to the positive effects of Cdc42, *C. albicans* Rac1 appears to downregulate Fre8-ROS (Figs. 6 and 7). This pattern of antagonistic functioning of two Rho GTPases in NOX ROS formation has previously been observed with both filamentous fungi (12) and mammals (37), although Rac activates and Cdc42 negatively regulates NOX ROS in these higher organisms (12, 37). The mechanism has been ascribed to Cdc42 competing with Rac for productive binding to NOX or to the NoxR/p67 regulator (12, 37). Interestingly, previous studies in *C. albicans* indicated that Rac1 may interfere with Cdc42 promotion of hyphal growth by titrating out an effector molecule (21). Fre8 may be one such effector of Cdc42 subject to Rac1 interference.

### Possible mechanisms for Cdc42 regulation of Fre8

**Fig. 10:**
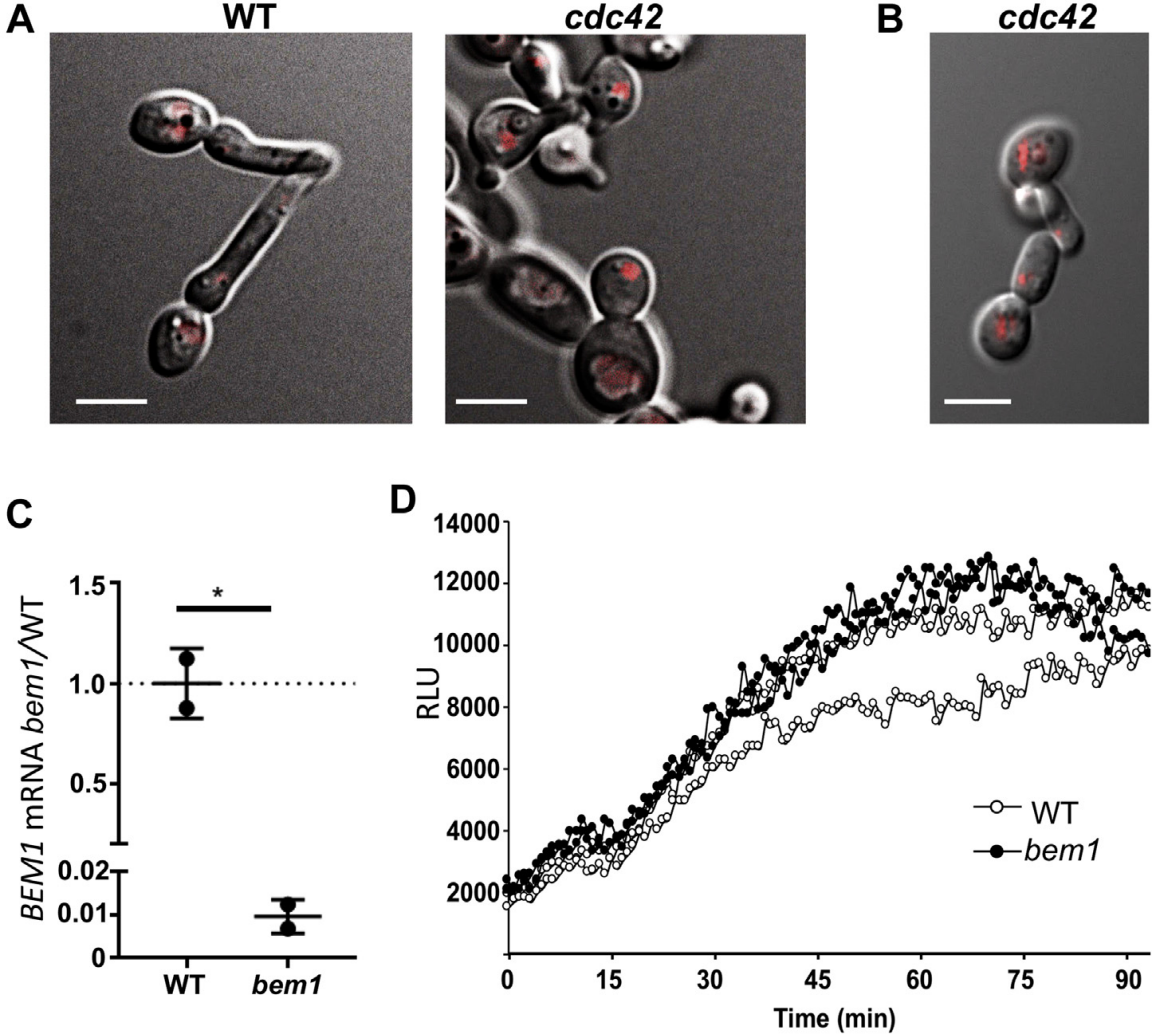
**Fre8-dTom expression and Fre8 ROS in mutants of *cdc42* and *bem1*.***A* and *B*, fluorescence microscopy of Fre8-dTom in *cdc42*-deficient cells induced to form germ tubes in the presence of methionine as for Figure 7. *A* and *B* are results from two independent experiments, and the bar represents 5 microns. *C* and *D*, a *Δbem1/*pMET*BEM1* strain subjected to methionine repression as for Figure 7 shows a ≈100-fold reduction in *BEM1* expression by quantitative RT-PCR (*C*) but no reduction in Fre8 ROS by luminol chemiluminescence (*D*). Results are from two independent cultures (*C*) and two experimental trials (*D*). ∗*p* = 0.015 as determined by two-tailed *t* test. The following strains were utilized: WT and *cdc42* as described for Figure 7; *bem1*, PY63. Fre8-dTom, Fre8 to dTomato; ROS, reactive oxygen species.

How might Cdc42 positively regulate Fre8 activity? Mutants of *cdc42* show no loss in *FRE8* mRNA levels (Fig. 7*B*), and by examination of Fre8-dTom, the NOX is present in *cdc42* mutants and localizes in the vacuolar network (Fig. 10, *A* and *B*). In *C. albicans*, active Cdc42 localizes exclusively at the hyphal tip during morphogenesis (38, 39), the same site as Fre8 ROS production. Therefore, Fre8 may be activated upon engaging Cdc42 or a Cdc42 effector molecule specifically at the hyphal tip. Such spatial control of NOX by Rho GTPases is characteristic of higher fungi and mammals: the bulk of NOX in these higher organisms is inactive in the vacuolar/lysosomal system and is only activated at locations where NOX contacts Rac or the NoxR/p67 regulator (7, 32).

In filamentous fungi and mammals, NoxR/p67 is central to NOX activation by Rac (6). As mentioned previously, yeast genomes lack NoxR/p67; however, they do express Bem1 that, in filamentous fungi, facilitates tethering of NoxR/p67 to membranes (8). In *C. albicans*, Bem1 is not critical for morphogenesis (19), and we observed that Bem1 is also not required for Fre8 activity during morphogenesis, as repressing *BEM1* expression by ≈100-fold failed to inhibit ROS production (Fig. 10, *C* and *D*).

Collectively, our studies demonstrate that in *C*. *albicans*, the Rho GTPase Cdc42 controls Fre8 ROS at the post-transcriptional level in the absence of NoxR/p67 and other accessory proteins used in Rac activation of NOX in higher fungi and mammals. The lack of a NoxR/p67 requirement may explain why *C. albicans* can use Cdc42 as opposed to Rac in the control Fre8 NOX. In higher fungi and mammals, Rac interactions with NoxR/p67 require Rac residues A27 and G30 that are absent in Cdc42 (6, 12, 37, 40, 41, 42). The only other known case of Rho GTPase control of NOX without NoxR/p67 occurs with plant NOX, and there, Rac regulation involves EF hands in the NOX polypeptide (43, 44). Fre8 contains no EF hands, so a distinct mechanism must be involved. As one possibility, *C. albicans* Cdc42 may directly activate Fre8 through physical interactions with the NOX. If so, this would represent a unique mechanism for Rho GTPase regulation of ROS, as *C. albicans* is missing the NoxR/p67 required for Rac activation of NOX in filamentous fungi and animals. Rac in these higher organisms physically interacts with NoxR/p67 (6, 8). The Bokoch group reports that Rac can also bind mammalian NOX2 (37, 40, 45), although this is somewhat controversial (6). In plants, Rac1 physically interacts with the EF hand containing region of plant NOX (43, 44), but since there are no EF-hand motifs in *C. albicans* Fre8, as well as no NoxR/p67 regulator, any direct interactions between Cdc42 and Fre8 would be unlike that of higher organisms. In addition, such physical interactions would represent a small pool of Fre8, as only a low level of Fre8 associates transiently with the apical cortex where active Cdc42 resides (Fig. 4). As an alternative possibility, Cdc42 may not directly interact with Fre8 but rather work through as-of-yet unknown regulators of Fre8 ROS, analogous to NoxR/p67. The effects of Cdc42 on Fre8 activity may also be indirect *via* a Cdc42 effector. There are numerous downstream targets of Cdc42 that are required for filamentation, including p21 and MAP kinases, formins, and others (22), and Fre8 NOX may fall under control of one of these effectors. Since Cdc42 control of Fre8 NOX is clearly post-transcriptional, the mechanism involving such effectors would be distinct from the recently reported transcriptional control of *S. cerevsiae* Yno1 NOX by MAP kinase pathways (46).

## Conclusion

Cdc42 has long been known to drive morphogenesis in *C. albicans*, and until now, the assumption has been that this control of polarized growth occurs without the ROS burst characteristic of multicellular eukaryotes. Our studies challenge this dogma and demonstrate that Rho GTPase–controlled polarized growth in unicellular yeasts can also involve post-transcriptional activation of NOX enzymes. To date, there have been two mechanisms ascribed for Rho GTPase control of NOX ROS. The first involves the large multisubunit NOX complex including the essential NoxR/p67 regulator that is the direct target of the Rho GTPase. The second involves Rho GTPase interaction with EF hands on NOX. Since Fre8 lacks EF hands, and since the essential regulatory subunits are missing in yeast genomes, Cdc42 control of Fre8 ROS must require a third distinct mechanism. This first report of a distinct alternative for Rho GTPase control of ROS is not likely unique to *C. albicans*. Other unicellular and pathogenic fungi have multiple *FRE8*-like genes (47) and may similarly use ROS for signaling polarized growth. Our studies will pave the way to more globally examine how unicellular fungi use ROS for growth and pathogenesis.

## Experimental procedures

### Yeast strains and growth conditions

The *C. albicans* mutant strains used in this study were derived from the clinical isolate SC5314 or from BWP17 (*ura3*::imm434*/ura3*::imm434 *iro1/iro1*::imm434 *his1*::hisG*/his1*::hisG *arg4/arg4*) or SN250 (*his1Δ/his1Δ, leu2Δ::Candida dubliniensis HIS1/leu2Δ::Candida maltosa LEU2, arg4Δ/arg4Δ, URA3/ura3Δ::imm434, IRO1/iro1Δ::imm434*). The *fre8Δ/Δ* strain CA-JG211 was constructed as described from SC5314 (26). The *Δcdc42*/pMet*CDC42* (PY47), *Δcdc24*/pMet*CDC24* (PY101), and *Δbem1*/pMet*BEM1* (PY63) strains were generated from BWP17 as published (19, 34), as was the *rac1Δ/Δ* (PY189) strain (21). The *rga2Δ/bem3Δ* 615 strain derived from BWP17 (36) was a gift from Alexandra Brand and Peter Sudbery. The SN250:*dck1Δ/Δ* strain was obtained through the Fungal Genetics Stock Center.

Strains expressing Fre8-dTomato were created using a plasmid containing the coding sequence of *FRE8* fused at the C terminus to dTomato, constructed from the pENO1-dTom-NATr vector (Addgene plasmid no. 52208; (48)) and kindly provided by Dr Julie Gleason. The plasmid was linearized within *ENO1* at *Not*1 and used to transform the *fre8Δ/Δ* null strain CA-JG211, BWP17, and the *Δcdc42*/pMet*CDC42* strain PY47. To express isolated dTomato, SC5314 was transformed in a similar manner with the backbone vector pENO1-dTom-NATr (Addgene plasmid no. 52208; (48)).

A strain expressing FRE8-GFP as a sole copy at its endogenous locus (PY6053) was constructed by sequential transformations, using primers CaFre8S1-AFP and CaFre8S2 (Table S1) initially with the pFA-GFPgamma-HIS1 plasmid (49) and the CaBWP17 strain (50) (PY4860) to generate PY5590 and subsequently with the pFA-GFPgamma-ARG4 plasmid (49) and PY5590 to generate PY6053. Correct integrants were verified by PCR for the presence of the *FRE8*-GFP fusion and the absence of endogenous *FRE8*, using in particular primer pairs CaFRE8p2076 and GFPm106 and CaFRE8p2076 and CaFRE8m2376, respectively (Table S1).

All strains were maintained at 30 °C in a yeast extract, peptone-based medium (yeast extract peptone dextrose [YPD]) with 2% (w/v) glucose. Studies involving morphogenesis utilized yeast-form cells obtained from log phase cultures, that is, culture absorbance of 1.0 to 2.0 at 600 nm. Typically, the yeast-form cells were obtained from YPD that in the case of BWP17 was supplemented with 80 mg/l uridine. In experiments involving methionine-repressible expression of *CDC42*, *CDC24*, and *BEM1*, yeast-form cells were obtained by growth in a synthetic complete medium containing 0.67% yeast nitrogen base and 2% glucose but lacking cysteine and methionine.

To induce morphogenesis, yeast-form cells were inoculated at an absorbance of 0.2 at 600 nm in either IMDM (Gibco; 21056023), in YPD + 10% fetal bovine serum (FBS; heat inactivated; Corning/Cellgro), or in the reaction mixture for luminol chemiluminescence (described later) containing 25% IMDM. With IMDM and serum induction, cells were first starved in water at 30 °C for 30 min prior to hyphal induction, and in all cases, morphogenesis proceeded for 37 °C at 1 h or for indicated time points. In experiments involving methionine-repressible expression of *CDC42*, *CDC24*, and *BEM1*, 2.5 mM of methionine was added in the final 2 h of yeast-form growth and during hyphal morphogenesis. In Figure 4, filamentous growth induction was carried out as described previously with 50% serum (21).

### Biochemical assays, microscopy, and statistical analysis

For luminol chemiluminescence analysis, cells were induced to form germ tubes for 1 h in IMDM as described previously, and 50 μl of this culture was added directly to a 96-well plate containing 150 μl of Hanks buffered saline solution (HBSS) with a final concentration of 0.15 mM luminol (Cayman Chemical Company) and 2.67 units per milliliter of horseradish peroxidase. In studies where yeast-form cells were added to the luminol reaction, 0.01 to 0.02 units of cells at an absorbance at 600 nm were added to the reaction mix containing 25% IMDM and 75% HBSS. The samples were analyzed for luminol chemiluminescence at 37 °C using a Biotek Synergy HT plate reader (Biotek) as previously described (26). To calculate total luminescence over a 90-min time interval, the area under the curve was calculated using GraphPad Prism 8 software (GraphPad) with background luminescence subtracted.

For quantitative RT-PCR analysis, germ tube cells were prepared in 20 ml IMDM as described previously. In experiments where yeast-form cells were induced to form hyphae during the course of the luminol assay, a 25-ml reaction containing 25% IMDM and 75% HBSS was used, and cells were harvested at designated time points. Pelleted fungal cells were washed twice with diethylpyrocarbonate-treated water, and RNA was prepared by the hot acid phenol method (51). Complementary DNA was prepared using the Maxima H Minus First Strand cDNA Synthesis Kit or the RevertAid cDNA Synthesis Kit (Thermo Fisher Scientific). Quantitative RT-PCR was completed using the Powerup SYBR Green Master mix (Thermo Fisher Scientific). Values were normalized to *TUB2* using the Δ*C*_*T*_ method, where *C*_*T*_ is the threshold cycle. Amplicons of ∼200 bp were obtained using the designated primers for *FRE8*, *TUB2*, *BEM1*, *CDC42*, and *CDC24* (Table S1).

NBT staining was carried out with germ tubes formed for 45 min in a 10-ml culture of FBS containing YPD. Cells were harvested, washed once in HBSS, and resuspended in 1 ml of a solution containing 0.05% NBT (w/v) in 25% IMDM and 75% HBSS. Cells were incubated at 37 ^o^C for 30 min at 220 rpm in the dark. Samples were harvested, washed once in HBSS and once in 70% ethanol, and resuspended in 200 ml 50% glycerol/HBSS prior to light microscopy at 100× magnification on a Zeiss Axio ImagerA2 microscope (Zeiss).

In studies of morphogenesis, dark field microscopy of live *C. albicans* cells was carried out using a Nikon Infinity 1 microscope at 40× magnification. Measurements of germ tube length were obtained using ImageJ at a ratio of 1.5 μm per 10 pixels. For fluorescence microscopy of Fre8-dTom of dTom-expressing cells, germ tubes were prepared as described previously from IMDM or in FBS. In cases where vacuoles were stained with CMAC (29), 5 μM CMAC (Thermo Fisher Cell Tracker Blue) in dimethyl sulfoxide was added from a 10 mM stock to cultures in the final 30 min of hyphal formation; 2.0 units at an absorbance of 600 nm were harvested and resuspended in 1 ml of sterile water; 10 μl of this cell suspension was mixed with 10 μl of fluoromount-G mounting medium and added to a microscope slide (Fisherbrand Superfrost Plus). No. 1 coverslips were added and sealed using nail polish. Fluorescence was imaged using the Zeiss Observer Z1 fluorescence microscope at 100× using either the DsRED channel for dTomato, the 4′,6-diamidino-2-phenylindole, channel for CMAC or differential interference contrast microscopy. Fre8-GFP cells were imaged with a spinning-disk confocal microscope as described (52); 16 × 0.4 μm z-sections were acquired using a 488 nm laser for GFP. Sum intensity projections of ten z-sections are shown, along with the central z-section. Line plot intensity profiles, with a three pixels line width, were generated from the sum projections, and image analyses were carried out using ImageJ 1.51 software.

With data analysis, differences were considered statistically significant when *p* values were ≤0.05 by applying either a two-tailed *t* test or a one-way ANOVA with a Tukey's post-test as determined by GraphPad Prism 7 software.

## Data availability

All data on ROS production, gene expression, and hyphal length are contained within this article. Additional cell images can be obtained upon request from V. Culotta (Figs. 2 and 3) and M. Bassilana (Fig. 4).

## Supporting information

This article contains supporting information.

## Conflict of interest

The authors declare that they have no conflicts of interest with the contents of this article.
