# Supplementary material for: Cdc42 regulates reactive oxygen species production in the pathogenic yeast Candida albicans
Source: J Biol Chem. 2021 Jun 25;297(2):100917. doi: 10.1016/j.jbc.2021.100917 (PMC8329510; doi:10.1016/j.jbc.2021.100917)
Supplement: Table S1 [file mmc1.pdf]

<u>Supporting Information</u>

Cdc42 regulates reactive oxygen species production in the pathogenic yeast *Candida albicans*

Griffin P. Kowalewski[1], Asia S. Wildeman[1], Stéphanie Bogliolo[2] Angelique N. Besold[1], Martine Bassilana[2] and Valeria C. Culotta[1]*

[1]The Department of Biochemistry and Molecular Biology, Johns Hopkins University Bloomberg School of Public Health, Baltimore, MD 21205 USA

[2]Université Côte d'Azur, CNRS, INSERM, Institute of Biology Valrose (iBV), Parc Valrose, Nice, France

List of materials

Table S1: Primers

Table S1: Primers

Primer Name	Purpose	Sequence
CaFre8S1-AFP	Construct Fre8-GFP strain	5'-GCAATGGTTGATGAATTACGTTGTCAAGTCATTAAAGTTA TAAATAATCCTGAAAAGAAAAGAGTTGATTTCTATGATCA ATTACAAGGCTGGAGTGGTGCTGGCGCAGGTGCTTC-3'
CaFre8S2	Construct Fre8-GFP strain	5'-CAATTTAAAAAAAAATCTATGTAAAGAACTAAAATCGATA ATTAAATGAGGAAAAAGGGGGAAGGGAGATGAGTGAGT GGGTATATTATATCTCATCTGATATCATCGATGAATTCGAG-3'
CaFRE8p2076	PCR analysis of Fre8-GFP integrant	5'-GGAAGAAGCTGGTAGAAGTATTGCC-3'
GFPm106	PCR analysis of Fre8-GFP integrant	5'-CCGGAGACAGAAAATTTG-3'
CaFRE8m2376	PCR analysis of Fre8-GFP integrant	5'-GGTGTAGCCACCAGAATGTTC-3'
FRE8-forward	qRT-PCR *FRE8*	5'-CTTTCCATCGTCATATTGCCAG-3'
FRE8-reverse	qRT-PCR *FRE8*	5'-GCTGTGCCCCAAATCATAAATG-3'
TUB2-forward	qRT-PCR *TUB2*	5'-GAGTTGGTGATCAATTCAGTGCTAT-3'
TUB2-reverse	qRT-PCR *TUB2*	5'-ATGGCGGCATCTTCTAATGGGATTT-3'
BEM1-forward	qRT-PCR *BEM1*	5'-CATCAAGGCGATCACAAGTC-3'
BEM1-reverse	qRT-PCR *BEM1*	5'-TTGGTGGAGTACCACTTCCTG-3'
CDC42-forward	qRT-PCR *CDC42*	5'-TCCCCAATCACCCAGGAA-3'
CDC42-reverse	qRT-PCR *CDC42*	5'-TGCAGCTACTATAGCCTCGTCAA-3'
CDC24-forward	qRT-PCR *CDC24*	5'-ACGTGATTCCGACCTGTCATTTA-3'
CDC42-reverse	qRT-PCR *CDC24*	5'-GATGGCGGGAACTGTGAGA-3'
